# Interactive Machine Learning-Based Multi-Label Segmentation of Solid Tumors and Organs

**DOI:** 10.3390/app11167488

**Published:** 2021-08-15

**Authors:** Dimitrios Bounias, Ashish Singh, Spyridon Bakas, Sarthak Pati, Saima Rathore, Hamed Akbari, Michel Bilello, Benjamin A. Greenberger, Joseph Lombardo, Rhea D. Chitalia, Nariman Jahani, Aimilia Gastounioti, Michelle Hershman, Leonid Roshkovan, Sharyn I. Katz, Bardia Yousefi, Carolyn Lou, Amber L. Simpson, Richard K. G. Do, Russell T. Shinohara, Despina Kontos, Konstantina Nikita, Christos Davatzikos

**Affiliations:** 1 Center for Biomedical Image Computing and Analytics (CBICA), University of Pennsylvania, 3700 Hamilton Walk, Philadelphia, PA 19104, USA;; 2 Department of Radiology, Perelman School of Medicine, University of Pennsylvania, 3400 Civic Center Boulevard, Philadelphia, PA 19104, USA;; 3 School of Electrical and Computer Engineering, National Technical University of Athens, 9 Iroon Polytechniou St, 15780 Athens, Greece;; 4 Department of Pathology and Laboratory Medicine, Perelman School of Medicine, University of Pennsylvania, 3400 Civic Center Boulevard, Philadelphia, PA 19104, USA; 5 Department of Radiation Oncology, Sidney Kimmel Medical College & Cancer Center, Thomas Jefferson University, 233 S 10th St, Philadelphia, PA 19104, USA;; 6 Computational Breast Imaging Group (CBIG), University of Pennsylvania, 3700 Hamilton Walk, Philadelphia, PA 19104, USA; 7 Department of Biostatistics, Epidemiology, and Informatics, Perelman School of Medicine, University of Pennsylvania, Philadelphia, PA 19104, USA;; 8 Penn Statistics in Imaging and Visualization Center (PennSIVE), University of Pennsylvania, 423 Guardian Drive, Philadelphia, PA 19104, USA; 9 Department of Biomedical and Molecular Sciences, School of Medicine, Queen’s University, 18 Stuart Street, Kingston, ON K7L 3N6, Canada;; 10 Department of Radiology, Memorial Sloan Kettering Cancer Center, 1275 York Avenue, New York, NY 10065, USA;

**Keywords:** image segmentation, magnetic resonance imaging, computer tomography, artificial intelligence segmentation, magnetic resonance imaging, computer tomography, artificial intelligence

## Abstract

We seek the development and evaluation of a fast, accurate, and consistent method for general-purpose segmentation, based on interactive machine learning (IML). To validate our method, we identified retrospective cohorts of 20 brain, 50 breast, and 50 lung cancer patients, as well as 20 spleen scans, with corresponding ground truth annotations. Utilizing very brief user training annotations and the adaptive geodesic distance transform, an ensemble of SVMs is trained, providing a patient-specific model applied to the whole image. Two experts segmented each cohort twice with our method and twice manually. The IML method was faster than manual annotation by 53.1% on average. We found significant (*p* < 0.001) overlap difference for spleen (Dice_IML_/Dice_Manual_ = 0.91/0.87), breast tumors (Dice_IML_/Dice_Manual_ = 0.84/0.82), and lung nodules (Dice_IML_/Dice_Manual_ = 0.78/0.83). For intra-rater consistency, a significant (*p* = 0.003) difference was found for spleen (Dice_IML_/Dice_Manual_ = 0.91/0.89). For inter-rater consistency, significant (*p* < 0.045) differences were found for spleen (Dice_IML_/Dice_Manual_ = 0.91/0.87), breast (Dice_IML_/Dice_Manual_ = 0.86/0.81), lung (Dice_IML_/Dice_Manual_ = 0.85/0.89), the non-enhancing (Dice_IML_/Dice_Manual_ = 0.79/0.67) and the enhancing (Dice_IML_/Dice_Manual_ = 0.79/0.84) brain tumor sub-regions, which, in aggregation, favored our method. Quantitative evaluation for speed, spatial overlap, and consistency, reveals the benefits of our proposed method when compared with manual annotation, for several clinically relevant problems. We publicly release our implementation through CaPTk (Cancer Imaging Phenomics Toolkit) and as an MITK plugin.

## 1. Introduction

Medical image segmentation is an important task in clinical and research environments [1–4], facilitating subsequent computational analyses, which depend on the accuracy of the segmentation [5,6]. Manual expert annotations are currently considered the gold standard, which tend to be tedious, time-consuming, and often have limited reproducibility [3], even with the assistance of various tools [4].

A plethora of fully automatic machine learning (ML) methods that can achieve state-of-the-art results have been proposed, but tend to face various challenges [7] that hinder clinical translation. Some of the most important challenges are generalization to unseen datasets and need for extensive expert corrections and refinements [4,8]. Interactive machine learning (IML) methods fill the void between manual and automatic approaches by allowing an operator to train a patient-specific model via quick and rough drawings, which then automatically segments the entire scan [9–11]. IML approaches provide the option for expedited refinements, and the final segmentation tends to get closer to the desired result as a function of the invested time.

Two popular tools offering IML functionality are ITK-SNAP [8] and 3D Slicer [12]. ITK-SNAP has seen success; however, it requires users to follow a complex protocol to achieve multi-label segmentation. The user first provides quick drawings for the different ROIs and trains a model. Afterwards and separately for each class, the user must place seeds and evolve a contour. 3D Slicer has tools for both interactive and automated methods using traditional techniques such as region-based statistical methods. Specifically regarding IML, 3D Slicer’s “grow from seeds” effect works using GrowCut, but it can only support one image as input, putting at a disadvantage for segmentation of complex structures, like glioblastoma regions, which typically requires combination of information from multiple co-registered images, such as FLAIR, T1, T2, and T1Gd. Deep learning can also be used for IML segmentation [13], but there has not been many successful methods in this category and it has only been demonstrated in simpler tasks. It is not simple to create and train methods that handle the diversity of biomedical image techniques, as well as the variable number of input channels.

For manual segmentation, apart from the aforementioned tools, the Medical Imaging Interaction Toolkit (MITK) [14] v2021.02 has a complete set of segmentations utilities, with some allowing for interaction by means of seed placement, however they are target towards segmentation of homogenous lesions and use only one image as input. On the other end of the spectrum, the current state-of-the-art for fully automatic medical image segmentation is nnU-Net [15], a self-configuring U-Net-based method [16], which surpassed most specialized existing approaches in 23 public datasets used in international biomedical segmentation competitions.

In this study, we propose an IML method leveraging adaptive geodesic distance (AGD) [17] maps alongside an ensemble of support vector machines (SVMs) that is agnostic to image type/dimensionality. We aimed to create a method that is easy-to-use, and supports multiparametric input images, in an effort to address obstacles that we identified were keeping interactive approaches from wider use, while also remaining fast and allowing the radiologist to control the decision-making. We systematically evaluated the performance of the proposed method against manual expert segmentation across different anatomical structures and image modalities. Evaluation endpoints comprised speed, spatial overlap agreement, and consistency between different time-points and raters.

## 2. Materials and Methods

### 2.1. Data

Experiments were approved by the Institutional Review Board (IRB) of the University of Pennsylvania (UPenn). Quantitative evaluation was based on public and private clinical data from four retrospective cohorts (spleen (3D-CT, *n* = 20/41, Medical Segmentation Decathlon [18]); breast tumor (2D-DCE-MRI, *n* = 50, multimodality trial at UPenn; NIH P01CA85484); lung nodules (2D-CT, *n* = 50/89, The Cancer Imaging Archive [19–21]); brain glioblastoma (3D-MRI, *n* = 20/335, BraTS’19 [3,4,22])). Cohort subsets were created, following random selection, to facilitate the exhaustive manual annotations described hereafter. The brain (11 males, 9 females: mean age = 62.84/64.36, age range = 44.82–77.48/39.64–77.09) and breast (female: mean age = 50.41, age range = 32.68–71.97) datasets were acquired from 2006–2014 and 2002–2006, respectively. The spleen (13 males, 7 females: mean age = 63.85/58, age range = 40–81, 48–68) and lung (34 males, 16 females) were acquired from 2000–2013 and 2004–2011, respectively. Age information was not available for the lung dataset. Ground truth segmentations were available for all datasets, except for lung which were created by a fellowship-trained, board-certified thoracic radiologist (S.K., 21 years of experience).

### 2.2. Proposed Segmentation Algorithm

**Figure 1.**
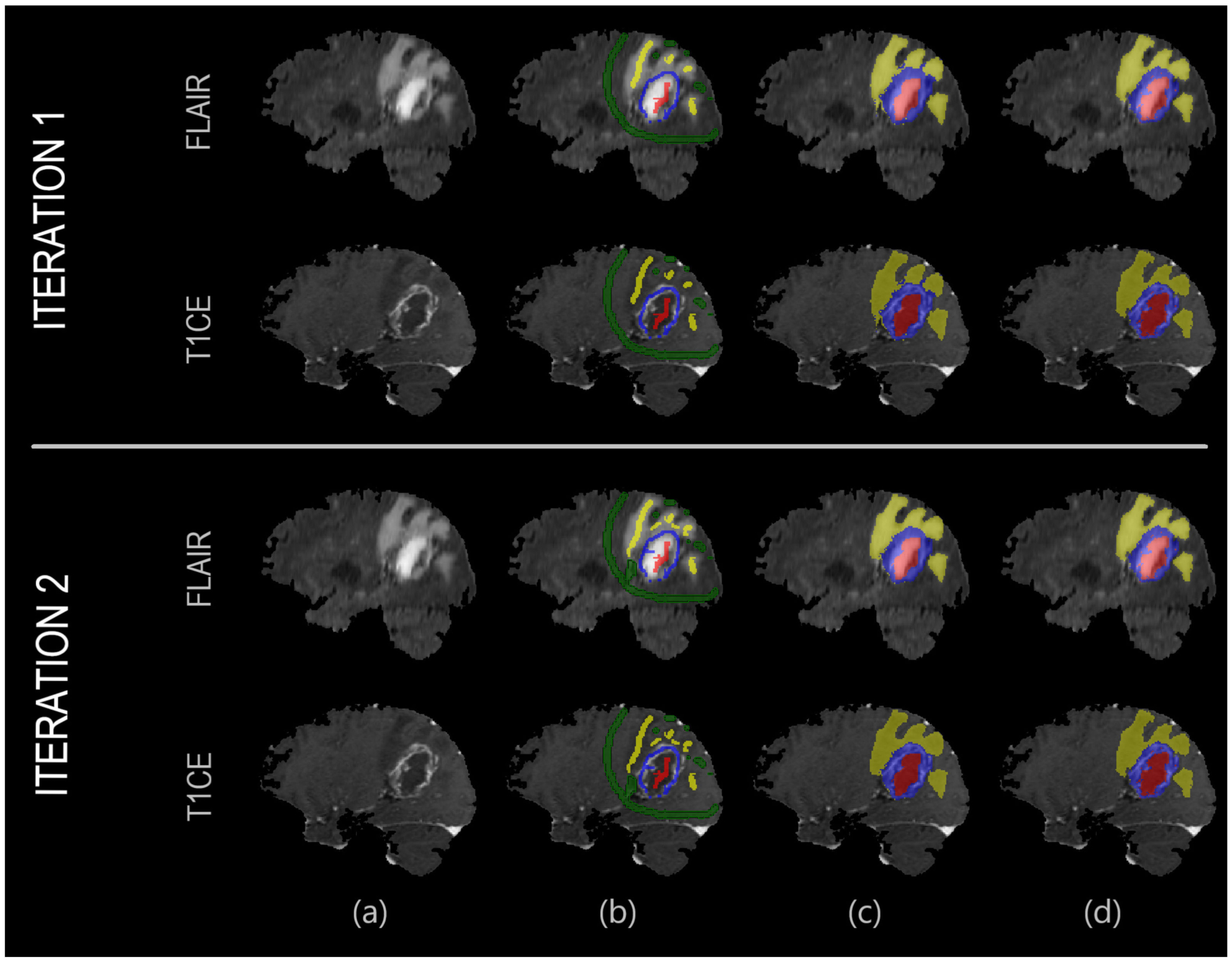
Example showcasing the result improving as a function of invested time. In the first iteration, the user quickly draws over the different areas. In the second iteration, the user places few additional labels to correct representative misclassified areas, which are then used to retrain the machine learning model. From left to right: (**a**) Anatomical image; (**b**) User annotations; (**c**) Result segmentation; (**d**) Ground truth segmentation.

The algorithm can segment N regions of interest (ROIs) at one time by initializing N + 1 different labels, where the additional one accounts for the “background”. As a first step, the user briefly draws over the different ROIs using distinct labels (Figure 1). All co-registered images are given to the algorithm as input. Every available co-registered sequence can be included; for instance, in brain tumor applications, this typically includes FLAIR, T1, T2, T1Gd.

Pre-processing is performed for anisotropic and/or large images. With a margin of 0.1 mm, if the input images have anisotropic spacing, i.e., the largest and the smallest voxel spacing values of an image are different by more than 0.1 mm, images are resampled to have the same spacing in all dimensions. The new selected universal spacing value is the lowest value, equal or higher than the lowest spacing value of the original images, that allows the resultant image to have less than 10 million voxels. The voxel number threshold was implemented for performance reasons. Likewise, if isotropic input images have more than 10 million voxels, they are resampled to the lowest spacing value, in all dimensions, that allows the voxel count to not exceed that limit. Resampling of labeled images is done using the nearest neighbor interpolation. The aforementioned resampling operations are part of the implementation and are not expected to be performed by the user. Results are always resampled back to the original image space. Lastly, all images are standardized to have 0 mean and 1 standard deviation.

**Figure 2.**
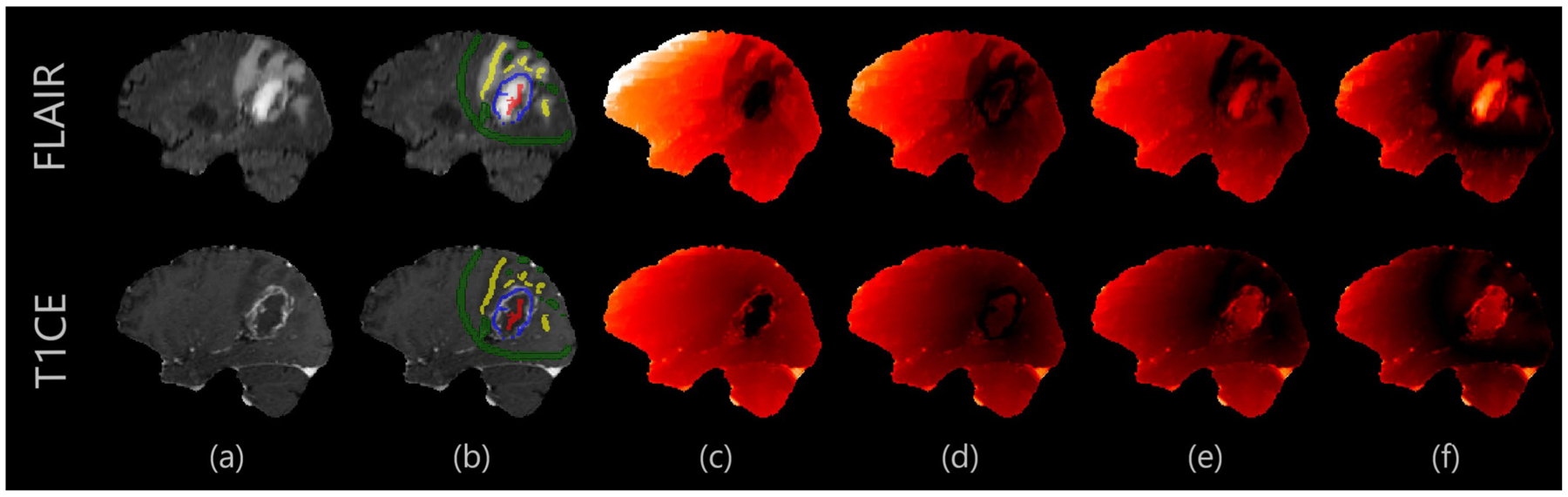
Example of AGD maps. Darker values indicate lower adaptive geodesic distance from the user drawings. In glioblastomas, non-enhancing tumor (NE) and enhancing tumor (ET) boundaries are clearer in T1CE, while the boundary between edema (ED) and background is clearer in FLAIR. From left to right: (**a**) Anatomical image; (**b**) User annotations; (**c**) Adaptive geodesic distance (AGD) map for NE annotation; (**d**) AGD map for ET; (**e**) AGD map for peritumoral edematous/infiltrated tissue (ED); (**f**) AGD map for background.

For each pair of image and class labels, an adaptive geodesic distance (AGD) map [17] (Figure 2) is produced reflecting a composite of intensity and spatial distance from the drawings, such that voxels far away and/or with very different intensity have higher values. The process is parallelized; each AGD map is created independently of each other. AGD maps are normalized in the [0, 1] range. To provide more spatial information, three “coordinate” maps are used, one for each dimension of the image, where the values range from 0 to the size of the image in that dimension.

Images are parsed using ITK [23] iterators and the image values are added to a two dimensional array (OpenCV’s [24] mat implementation is used), where size across the first dimension is the number of pixels/voxels in an image and across the second is the number of co-registered images. Likewise, pixels/voxels of the labeled image are added to a one-dimensional array. Only labeled samples are used for training. For performance reasons, if the number of labeled samples exceeds 3000, a balanced, i.e., retaining the ratio of samples per class, subset of 3000 labeled samples is used for training. Lastly, the selected training and labeled data are added to OpenCV’s “TrainData” structure, which is the format expected by the OpenCV’s machine learning implementations.

An ensemble [25] of SVMs is trained on voxels that belong to the drawings and segments the remainder of the image. Each training sample (i.e., voxel) is described by the following features: (i) intensity across all co-registered images, (ii) distance in all AGD maps, and (iii) value in all coordinate maps. Three SVM classification models (i.e., radial basis function (RBF), chi-squared, histogram intersection kernels) are trained in parallel and their hyperparameters are selected through cross-validation, using OpenCV’s [24] default grid search for optimizing the hyperparameters. Each voxel’s final prediction is obtained by fusing the three model predictions via majority voting and the RBF classifier is used to resolve ties.

Focusing on reproducibility, user-friendliness, and minimization of user interaction, we integrated the method to the Cancer Imaging Phenomics Toolkit (https://www.cbica.upenn.edu/captk, last accessed 11 August 2021) (CaPTk) [26] and as an MITK [14] plugin (https://github.com/CBICA/InteractiveSegmentation, last accessed 11 August 2021).

### 2.3. Experimental Design

#### 2.3.1. The Protocol Provided to Experts

**Figure 3.**
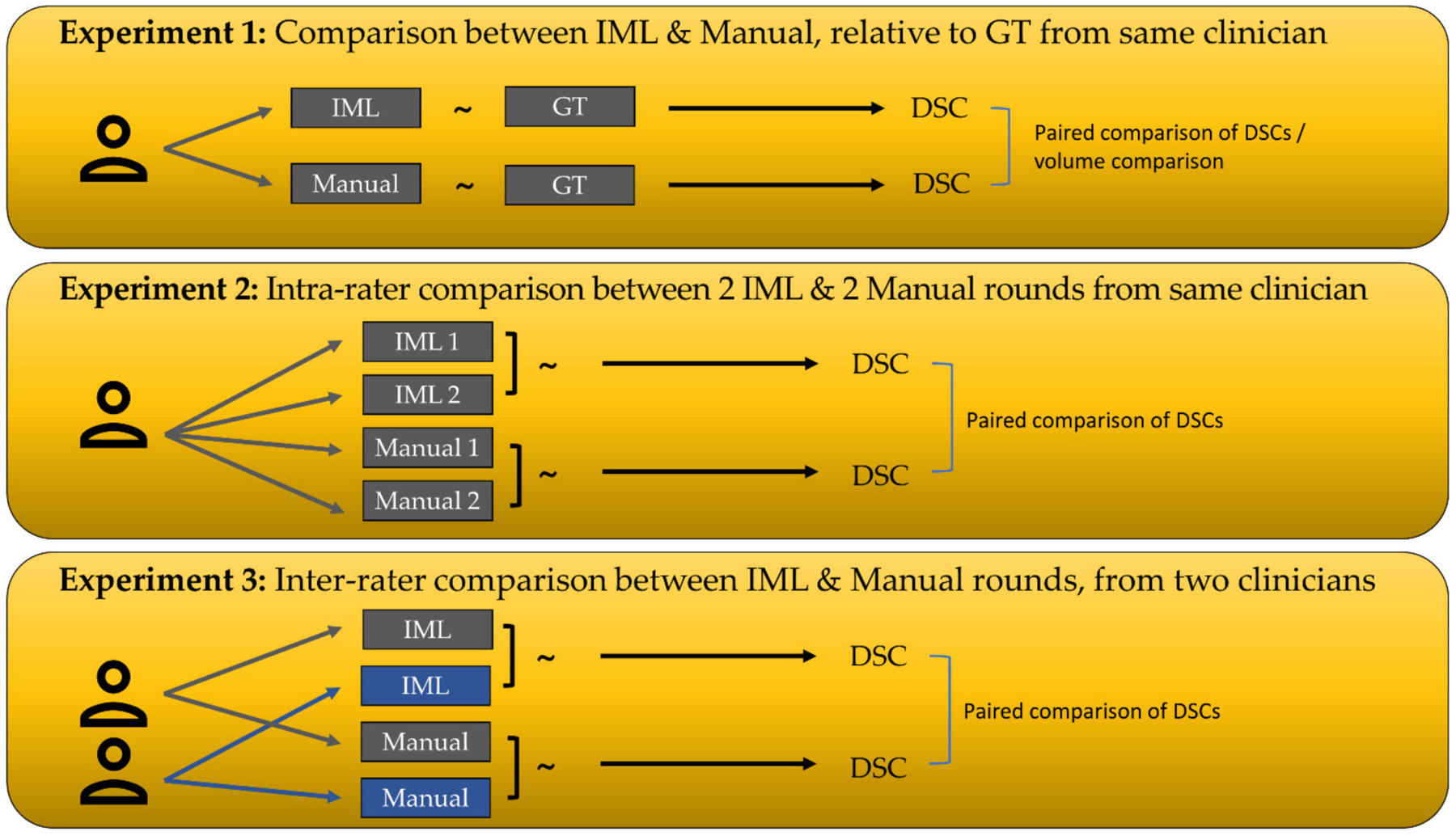
Summary of the main experimental design. The “~” symbol denotes calculation of Dice coefficient between two segmentations. DSC = Dice Similarity Coefficient, GT = Ground Truth.

To quantitatively evaluate our method, we included eight experts, two for each cohort. Each expert was asked to segment every scan four times, thereby producing two manual and two IML-assisted annotations, in addition to the extensively defined and verified ground truth (GT) segmentations. The experts were given brief instructions for our method and were asked to note the time needed for their segmentations. To have a fair assessment of inter-rater consistency for glioblastoma segmentation, we instructed the experts to perform the manual segmentation of the various tumor sub-regions (enhancing tumor (ET), non-enhancing tumor (NE), and peritumoral edematous/infiltrated tissue (ED) [3,4]) in 1 h or less. Figure 3 outlines the experimental design.

#### 2.3.2. Experiment 1—Overall Performance Evaluation

We initially evaluated the spatial overlap agreement of each approach relative to the ground truth by utilizing the Dice Similarity Coefficient (DSC) as a metric to select one IML-assisted and one manual segmentation from each rater. For glioblastoma, only the whole tumor (WT) area was used for these selections. The DSCs of the two sets were statistically compared. Additionally, the volumes calculated for IML and manual segmentations were quantitatively compared with the ground truth, by plotting volume pairs in scatterplots. Each pair comprise the volume of the approach and the volume of the ground truth for a particular case. Using regression, a line can be drawn from the IML-ground truth pairs and another one from the manual-ground truth ones. The closer these lines are to the middle line, that splits evenly the quadrant, the more similar the approach prediction volumes are to the ground truth ones and the closer to parallel the lines are, the more systematic were the potential errors. Furthermore, we estimated the Pearson’s Correlation Coefficient [27] for each of the paired segmentations: (i) IML correlation to ground-truth and (ii) manual correlation to ground truth. The average active drawing time, i.e., time spent on inspecting images and drawing input annotations, was compared for each cohort between IML-assisted and manual segmentation.

#### 2.3.3. Experiment 2—Intra-Rater Segmentation Consistency

The DSCs between the two IML-assisted and the two manual segmentations of each rater were calculated (i.e., DSC_IML1/IML2_, and DSC_Manual1/Manual2_) for each case. The DSCs were statistically compared. In addition, the existence of significant differences between the DSCs of the manual and IML-assisted segmentations relative to ground truth (i.e., DSC_IML/GT_, and DSC_Manual/GT_) were also statistically compared for each rater separately, to see how many raters were consistent using our method and how many when doing manual segmentations.

#### 2.3.4. Experiment 3—Inter-Rater Segmentation Consistency

The best segmentations of each rater were selected with the same selection criteria as Experiment 1. Raters were blind to each other’s segmentations. The DSCs between the best IML-assisted segmentations across raters, and between their best manual annotations were calculated for each case (i.e., DSC_IML Rater 1/IML Rater 2_, and DSC_Manual Rater 1/Manual Rater 2_), and their significant differences were evaluated.

#### 2.3.5. Statistical Analysis

We used paired Wilcoxon-signed rank non-parametric statistical tests [28] for statistical comparisons (assuming a type I error rate of 0.05), because the samples were paired and tended not to follow a Gaussian distribution. We used Python’s SciPy 1.4.1 package to perform the tests [29].

## 3. Results

In this section, the results of the experimental validation are presented for 20 spleen, 50 breast tumor, 50 lung tumor, and 20 glioblastoma cases.

### 3.1. Experiment 1: Overall Performance Evaluation

**Figure 4.**
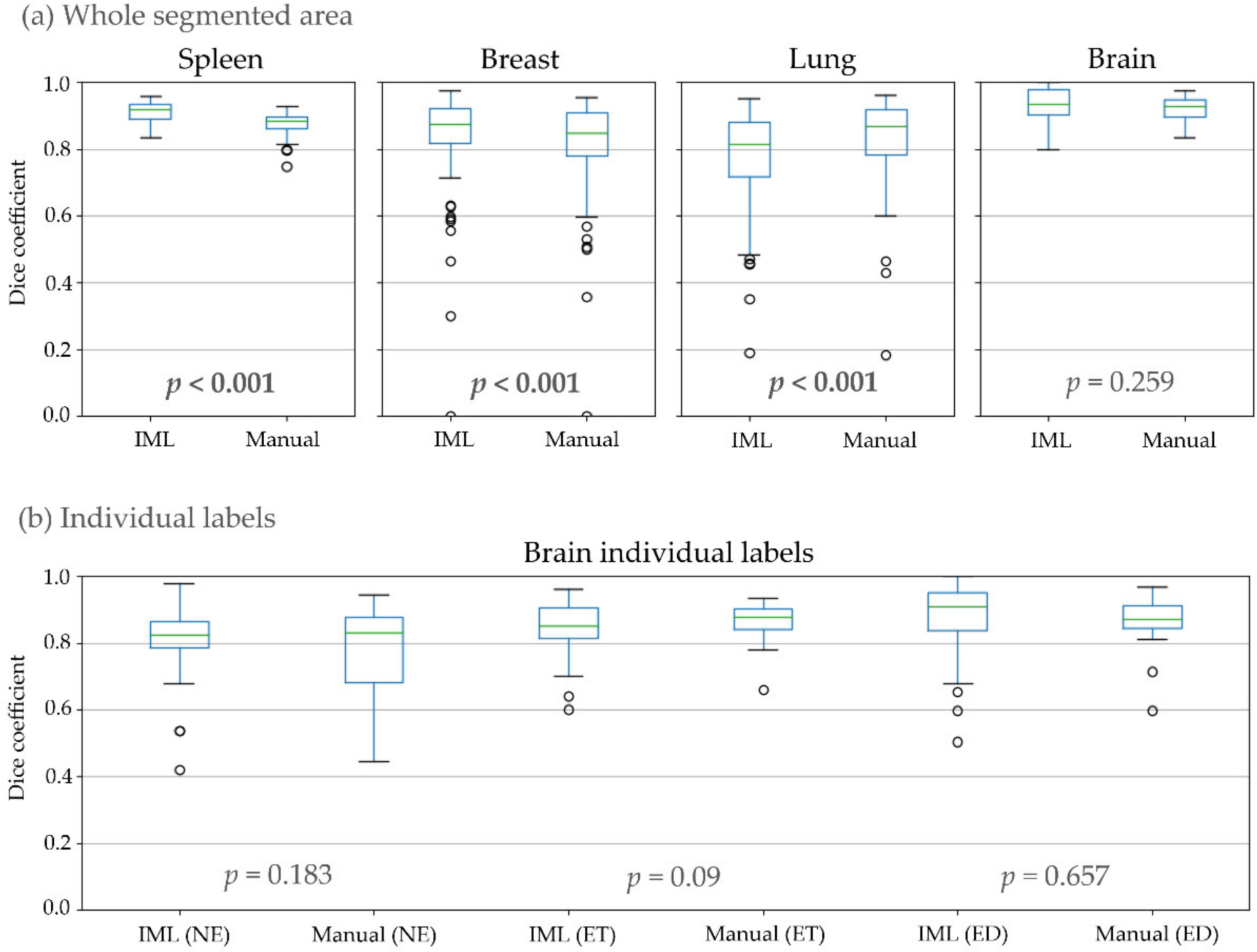
Dice coefficient, compared to ground truth, where: (**a**) All individual labels representing different areas of the structure counted as one; (**b**) Individual areas of glioblastomas.

In the first experiment, the performance of the proposed method was evaluated (Table 1, Figure 4). For glioblastomas, manual and IML-assisted segmentations yielded similar pairs of DSCs both for WT and individual sub-regions, thereby indicating no significant difference between them, whereas the converse was true for other cohorts. Our method achieved higher DSC on average for spleen and breast tumors, but lower for lung nodules when compared with the manual segmentations. However, our method was substantially faster than manual annotation in all cohorts (Table 1) by 53.1% on average.

**Figure 5.**
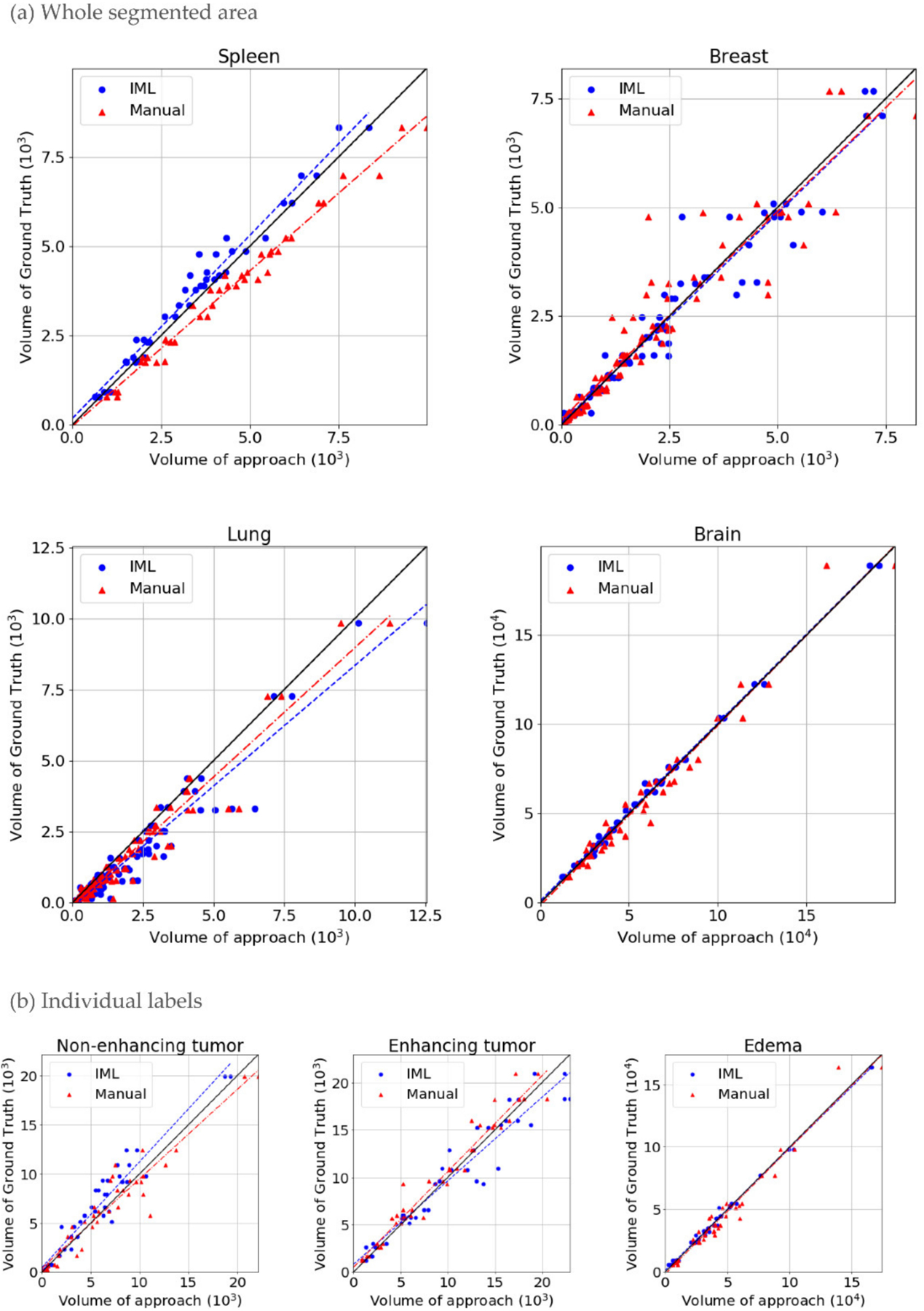
Scatterplots in which blue points are the pairs of volume of Interactive ML (IML) method and volume of ground truth and red are the pairs of manual segmentation volume and ground truth. The black line represents the ground truth’s volume. The plots belong to (**a**) Different cohorts where all individual labels, representing different areas of the structure, are counted as one; (**b**) The sub-regions of glioblastomas.

An analysis of the volume of ground truth, manual and IML-assisted segmentations (Figure 5, Table 1 “Correlation coefficient” column) shows that errors made by our method were mostly systematic. This is more evident in spleen images, where IML-assisted and manual segmentations revealed systematic under- and over-segmentation, respectively. Our method made some non-systematic errors in lung nodules and the ET glioblastoma sub-region, but these areas were also more erroneous in manual segmentations. Notably, ET is regarded as the most challenging area of glioblastoma, because it frequently has unclear and smooth boundaries [3].

### 3.2. Experiment 2: Intra-Rater Segmentation Consistency

**Figure 6.**
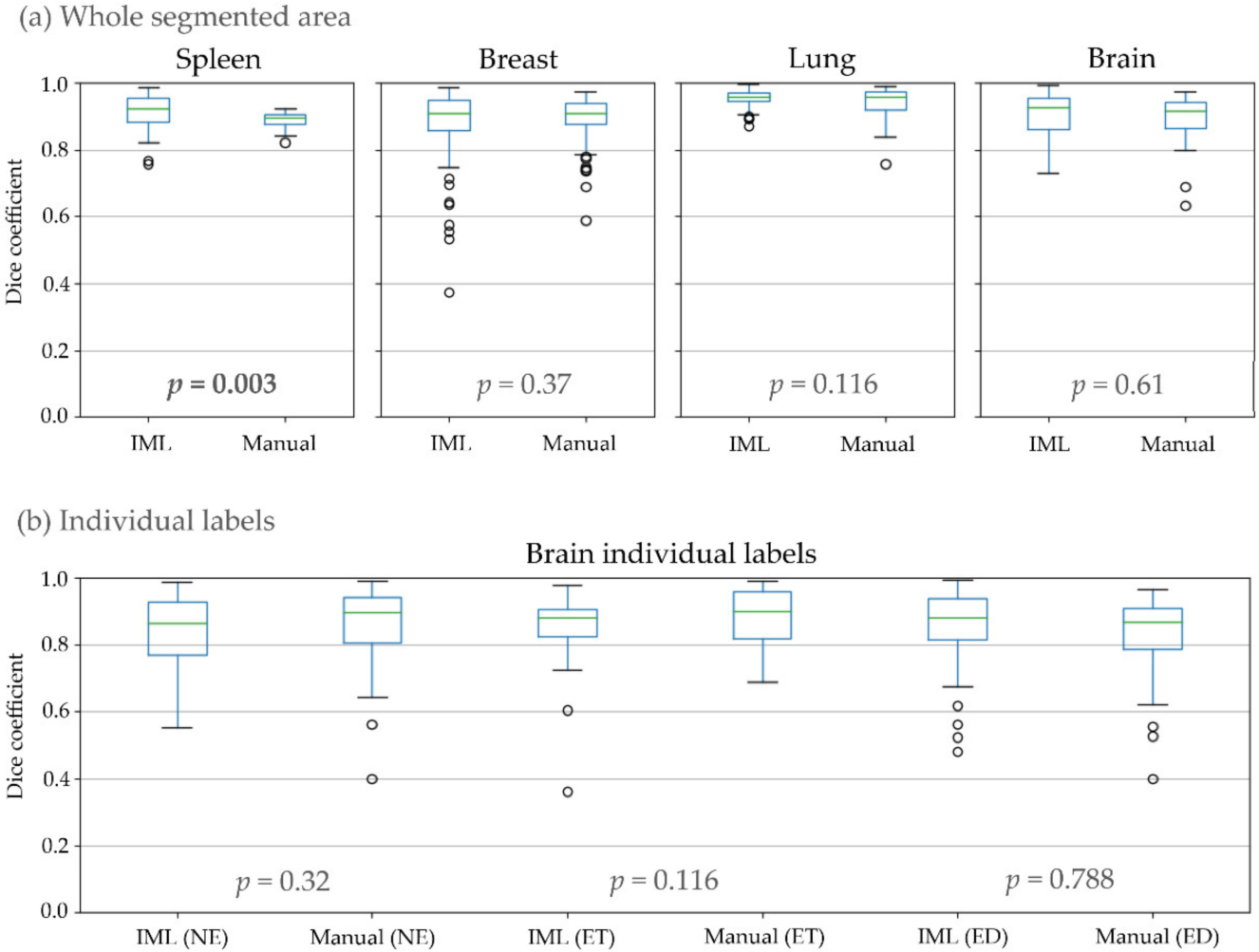
Dice coefficient between the first and second round of the raters where: (**a**) All individual labels representing different areas of the structure counted as one; (**b**) Individual areas of glioblastomas.

The second experiment attempts to quantify intra-rater consistency, comparing the two cycles of segmentations of each rater, separately for IML and manual (Table 1, Figure 6). No significant difference was found between manual and IML-assisted segmentations for any of the cohorts, except spleen where segmentations using our method had higher mean overlap.

Additional analysis of DSC relative to ground truth (Table 2) found a significant difference in only one of the raters when using the IML method, while revealing a significant difference in 4/8 raters for manual annotations. Furthermore, there was no significant difference when using the IML method for any of the two raters for individual sub-regions of glioblastoma. The same tests for manual annotations revealed a significant difference in all sub-regions, except ET in one of the raters.

### 3.3. Experiment 3: Inter-Rater Segmentation Consistency

**Figure 7.**
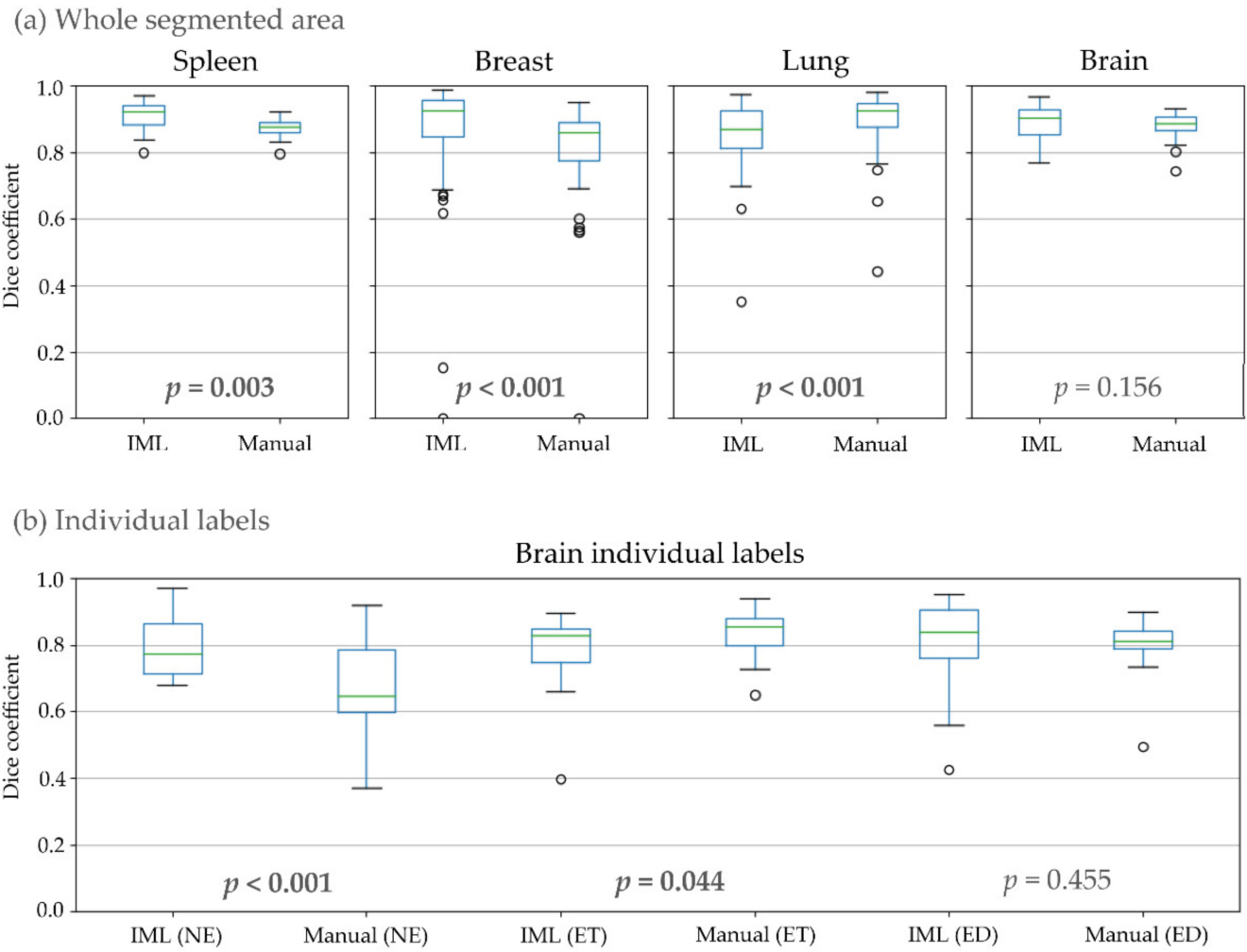
Dice coefficient, for inter-rater consistency, between segmentations of different raters, where: (**a**) All individual labels representing different areas of the structure counted as one; (**b**) Individual areas of glioblastomas.

In the last experiment, inter-rater consistency of IML and manual segmentations was calculated and compared (Table 1, Figure 7). There was a significant difference for spleen, breast, lung, and the NE and ET glioblastoma sub-regions. From those, our method achieved a higher overlap for spleen, breast, and the NE glioblastoma sub-region. Conversely, manual segmentations had higher agreement for lung and the ET region.

## 4. Discussion and Conclusions

In this study, we presented a general-purpose, easy-to-use, and fast IML-based segmentation method that can be applied in a multitude of research applications without requiring any adaptations to different domains or training of users. The method takes as input co-registered images and user drawings, to create AGD maps and train an ensemble of SVMs, used for segmenting the whole scan. We evaluated our method’s performance on solid structures across different cohorts, image modalities, and anatomical sites.

Our method utilizes the power of ML; however, it mitigates one of its known weaknesses, i.e., the need for extensive training and lack of reproducibility on new datasets. By virtue of being trained interactively, our segmentation models are optimal for the specific individual’s scans. Additional benefits include the ability of the method to be parallelized and low hardware requirements. The disadvantage of this approach is that it is not fully automated.

Our quantitative evaluation showed great promise for the applicability of the method in various structures relevant to medical research. Accuracy and inter-rater agreement were comparable to manual segmentation, while intra-rater agreement was high, indicating that the method is stable. Volumetric errors were mostly systematic, indicating that results can be improved through further iterations or volumetric operations like shrinking/expanding. Multiparametric image support allows the method to be used in more complex applications. The method was also shown to be fast and not require excessive interaction, which when combined with the low amount of training given to the clinical experts shows that the goal of creating an easy-to-use method was achieved. According to the evaluation of ITK-SNAP [11] on a subset of the BraTS dataset, in the glioblastoma regions, our study also evaluated, particularly, ET (Dice_IML_/Dice_ITK-SNAP_ = 0.85/0.69) and WT (Dice_IML_/Dice_ITK-SNAP_ = 0.94/0.85), and ITK-SNAP had a lower mean agreement with the ground truth. Time spent by users on inspecting images and drawing input annotations was also lower (Time_IML_/Time_ITK-SNAP_ = 21 min/27.8 min), while our methodology was significantly less complex.

Future research can improve this method on multiple fronts. Advanced ML techniques, such as semi-supervised learning, can potentially increase the accuracy and consistency of the results. Transfer learning could expand the range of tasks to non-solid structures, such as brain lesions. If a specific task is targeted, pre-trained population-derived models, atlases, and specialized preprocessing techniques can potentially aid in producing better segmentations. Furthermore, a prospective dataset, especially one acquired under different acquisition settings, would lend further validity to our method.

The results showed that our method has accuracy and inter-rater consistency on par with manual segmentation across different solid anatomical structures and modalities. Additionally, our method showed high intra-rater consistency and minimized user interaction.

## Figures and Tables

**Table 1.** Results for all three experiments. Experiment 1: Overlap is calculated as dice coefficient relative to ground truth. *p*-values are the result of paired comparisons between the highest scoring interactive ML-assisted and manual segmentations for each rater and each case. Correlation coefficient is calculated between the resultant and ground truth volumes. Experiment 2: Values indicate overlap between the first and second cycle of each rater. *p* < 0.05 indicates a significant difference between the results of interactive ML and manual segmentations. Experiment 3: Values indicate overlap between segmentations of different raters. *p* < 0.05 indicates a significant difference in the inter-rater results for the respective cohort.

	Experiment 1							Experiment 2			Experiment 3		
	Mean Overlap with Ground Truth			Mean Active Drawing Time		Correlation Coefficient		Mean Intra-Rater Overlap			Mean Inter-Rater Overlap		
Label	IML	Manual	*p*	IML	Manual	IML	Manual	IML	Manual	*p*	IML	Manual	*p*
						**Spleen**							
-	0.91	0.87	<10^−3^	66 s	100 s	0.99	0.99	0.91	0.89	0.003	0.91	0.87	0.003
						**Breast**							
-	0.84	0.82	<10^−3^	19 s	70 s	0.98	0.95	0.88	0.9	0.37	0.86	0.81	<10^−3^
						**Lung**							
-	0.78	0.83	<10^−3^	93 s	125 s	0.96	0.97	0.96	0.95	0.116	0.85	0.89	<10^−3^
						**Brain**							
WT	0.94	0.92	0.259	21m	60 m	1	0.98	0.91	0.89	0.61	0.89	0.88	0.156
NE	0.81	0.79	0.183			0.97	0.95	0.85	0.86	0.32	0.79	0.67	<10^−3^
ET	0.85	0.87	0.09			0.96	0.98	0.85	0.88	0.116	0.79	0.84	0.044
ED	0.88	0.87	0.657			1	0.98	0.85	0.83	0.788	0.81	0.8	0.455

**Table 2.** Results (*p*-values) of a paired Wilcoxon test for each rater, comparing the dice coefficient results of the different approaches relative to ground. *p* < 0.05 indicates a significant difference.

	Rater 1		Rater 2	
Label	IML	Manual	IML	Manual
	**Spleen**			
-	0.9405	0.3507	0.1454	0.433
	**Breast**			
-	0.2425	0.5116	0.5921	0.1358
	**Lung**			
-	0.0422	<0.0001	0.1358	<0.0001
	**Brain**			
WT	0.156	0.0001	0.8813	0.0008
NE	0.0522	0.0859	0.9405	0.0001
ET	0.1913	0.3317	0.0522	0.0001
ED	0.4781	0.0001	0.6274	0.0008

## Data Availability

No data is made available, except the long nodule data which is a public dataset. All software is freely available.

## Featured Application:

The proposed interactive segmentation method can be used to facilitate faster and consistent creation of annotations for large-scale studies, to enable subsequent computational analyses. The proposed method combines strengths of expert-based annotations and machine learning.
